# Mechanobiology of gastric needle insertions: a combined experimental and numerical study

**DOI:** 10.1007/s10237-025-01986-z

**Published:** 2025-08-13

**Authors:** Sif Julie Friis, Torben Strøm Hansen, Mette Poulsen, Peter Helding Kvist, Ansgar Petersen, Hans Gregersen, Jens Vinge Nygaard

**Affiliations:** 1 Department of Biological and Chemical Engineering, Aarhus University, Gustav Wieds vej 10, 8000 Aarhus C, Denmark; 2 Alternative Delivery Technologies, Device and Delivery Solutions, Novo Nordisk A/S, Hilleroed, Denmark; 3 Device and Delivery Solutions, Novo Nordisk A/S, Hilleroed, Denmark; 4 Safety Pharmacology and Early Toxicology, Novo Nordisk A/S, Maaloev, Denmark; 5 Berlin Institute of Health at Charité, BIH Center for Regenerative Therapies, Berlin, Germany; 6 California Medical Innovations Institute, San Diego, CA USA

**Keywords:** Drug delivery device, Gastric tissue mechanics, Mechanobiology, Needle insertion, Numerical analyses, YAP-1

## Abstract

The rising use of biologic drugs has increased the demand for alternative gastric administration methods. Inception of devices engineered to insert medication into the mucosal lining overcomes limitations of traditional administration methods. Mechanical forces from such microneedle insertions can affect tissue and cellular behavior, particularly mechanotransduction markers. This study investigates the effects of needle insertion in gastric tissue to inform the design of alternative drug delivery devices. Experimental and computational approaches were utilized, using tension and radial compression tests on porcine gastric tissue to inform a finite element analysis (FEA) model. This model was validated with atomic force microscopy (AFM)-based micro-indentation to examine stiffness variations near the insertion site, and yes-associated-protein-1 (YAP-1) expression was analyzed to assess cellular mechanotransduction. AFM results revealed a distance-dependent decrease in tissue stiffness from the insertion site (*p* < 0.05), with significant differences in needle geometry (*p* < 0.05). The FEA model correlated well with AFM findings, confirming its validity for further cellular simulations. Mechanical stresses from needle insertion were shown to propagate through the tissue, affecting both cytoplasmic and nuclear stress distributions and altering nuclear morphology near the insertion site. The blunt needle produced a higher localized stress field compared to the sharp needle. Additionally, YAP-1 expression was lower in the injected samples than in control samples showing distance-dependent responses observed. This study demonstrates a validated model linking tissue mechanics and cellular responses, highlighting how needle geometry impacts gastric tissue mechanics and mechanotransduction, providing insights essential for designing gastric drug delivery devices.

**Supplementary Information:**

The online version contains supplementary material available at 10.1007/s10237-025-01986-z.

## Introduction

The market of biologic drugs, i.e., commercial therapeutic molecules like peptides and proteins, is rapidly developing. With health areas such as rheumatoid arthritis, cancer, and diabetes, the market value of the biologics is expected to reach US$ 400B by 2025 (Urquhart 2019). Despite this significant development, the delivery of drugs has limited their use. As such, patients are typically required to administer the biologic drugs intravenously or subcutaneously instead of oral delivery due to degradation in the gastrointestinal environment and limited permeability across biological barriers (Moroz et al. 2016; Anselmo et al. 2019). Research has demonstrated that most patients find orally administered medications more convenient (Guerci et al. 2019). Moreover, biologics are generally considered to have a better safety profile compared to small molecules due to their higher target specificity, reducing the likelihood of off-target effects (Poduval et al. 2023). Hence, it is of significant interest to investigate the potential of delivery of such pharmaceuticals in an oral format. The healthcare industry is undergoing a paradigm shift prompted by the imperative to surmount challenges inherent in the gastrointestinal (GI) tract and address the escalating demand for alternative technologies (Caffarel-Salvador et al. 2017). This is exemplified by the inception of devices engineered to insert the medication into the mucosal lining of the small intestine and stomach by needle insertion, thereby overcoming the limitations associated with traditional administration methods (Caffarel-Salvador et al. 2017; Verma, et al. 2019; Abramson et al. 2022a; Abramson et al. 2019a, b, b; Bellinger, et al. 2016; Liu et al. 2017; Dhalla et al. 2022; Kirtane et al. 2018; Hashim et al. 2019; Traverso et al. 2015).

The field of oral delivery devices remains in its early stages of development. Consequently, needle insertions into gastric tissue through an experimental and numerical setup has previously been investigated in a preceding study (Friis et al. 2025). The goal of that study was to potentially ease the early stages of device development and the associated parameter evaluation. Therefore, that study focused on passive tissue properties and the needle insertion itself by addressing the impact of needle geometry and insertion velocity on penetration forces, as well as stress and displacement fields (Friis et al. 2025). In the current study, it is aimed to further study the subject of needle insertion into gastric tissue from a passive mechanical and spatial cellular point of view, i.e., a mechanobiological perspective.

Among devices engineered to overcome traditional administration limitations, is the SOMA device (Abramson et al. 2019b). The current study specifically focuses on this device, thus, merely analyzing samples from the antral stomach region. The reason here fore is that patients are instructed to ingest SOMA on an empty stomach and in an upright position. This ensures the device localizes to the stomach antrum lining, orients its injection mechanism toward the tissue wall, and injects a drug into the gastric wall (Abramson et al. 2019b).

A needle insertion through structural tissue layers causes a local shift in the underlying strain field, resulting in permanent changes to the tissue as residual stress is released (Dou et al. 2006; Gregersen 2000). This mechanical perturbation is assumed to influence the surrounding cellular environment, triggering intracellular responses. Mechanosensitive cells adjust their structure and function in reaction to the changes in the mechanical environment, including the changes in stress and strain fields induced by needle insertion (Janmey et al. 2013). These mechanical changes are fundamental to understanding how tissues and cells respond to external forces, such as those encountered during medical procedures.

Mechanobiology research has shown that mechanical forces, whether endogenous or externally applied, profoundly impact cellular behavior. Cells sense and respond to mechanical cues, e.g., through changes in changes in shape, orientation, focal adhesion maturation, cytoskeletal force generation, cell organization during tissue formation, and ECM reorganization. These processes are key to biological events like tissue remodeling, wound healing, and development. Local mechanical forces, modulated by tissue stiffness and microstructural geometries, activate intracellular signaling pathways, modulating gene expression, and cellular function (Elosegui-Artola et al. 2017; Huang et al. 2023; Panciera et al. 2017). In this context, yes-associated protein 1 (YAP-1), a transcriptional regulator, plays a pivotal role in transducing mechanical stimuli into cellular responses. YAP-1 regulates gene expression in response to mechanical signals, with its localization within the cell serving as an indicator of mechanical force exposure (Janmey et al. 2013; Elosegui-Artola et al. 2017; Huang et al. 2023; Panciera et al. 2017; Dupont et al. 2011; Scott et al. 2021; Novev et al. 2021; Pocaterra et al. 2020; Poh et al. 2012). Research indicates that mechanical forces, including hydrostatic pressure and localized indentation, affect YAP-1 expression, leading to an increase in its nuclear presence in response to mechanical stimuli (Poh et al. 2012; Al-Nuaimi et al. 2024). Furthermore, YAP-1 expression is linked to changes in nuclear curvature and pore membrane dynamics, indicating its role as a sensor of mechanical cues in the cellular microenvironment (Ghagre et al. 2024; Zimmerli, et al. 2021; Venturini, et al. 2020). This makes YAP-1 an ideal biomarker for studying cellular responses to mechanical perturbations.

It is hypothesized that the presence of a needle in the gastric tissue induces localized compression in the surrounding tissue, leading to altered mechanical properties and potentially affecting cellular behavior.

Here, we present the aim of the study as a dual work: (1) developing and experimentally validating a numerical model of needle insertion into gastric tissue, in which both bulk mechanical properties of individual tissue layers and cellular properties are incorporated, and (2) identifying the impact of needle insertions on gastric biomechanical and cellular properties bridging length scales down to the single cell level.

## Materials and methods

### Material characterization (uniaxial tension and radial compression)

Experimental tensile and radial compression tests were performed to characterize anisotropic and hyperelastic tissue properties. This was done and published in a previous study. The previous study detailed the experimental setup, handling, and preparation of the gastric tissue (Friis et al. 2023).

**Fig. 1:**
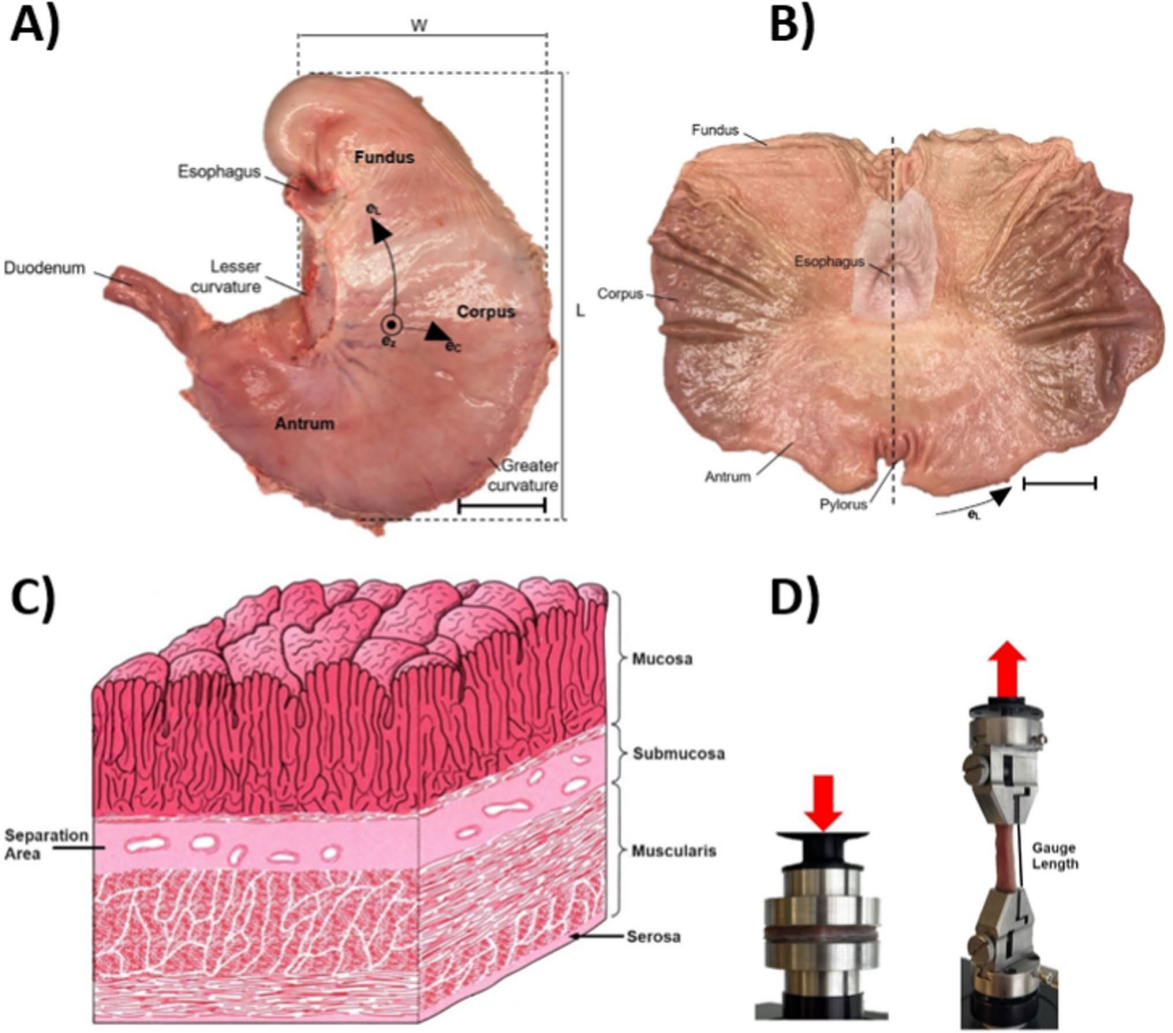
Anatomical and experimental characterization of the porcine stomach. **A** Front view of the porcine stomach showing key anatomical regions, including the fundus, corpus, and antrum. The black arrows and labels indicate the circumferential and longitudinal directions, length, L, and width, W. Scale bar 50 mm. **B** View of the opened stomach, highlighting the regions from inside. Scale bar 50 mm. **C** Illustration of the stomach layers, depicting the mucosa, submucosa, muscularis, and serosa. The illustration is edited with approval from the original authors and publisher (Geneser et al. 2020). **D** Experimental setup for mechanical testing: radial compression test (left) and uniaxial tensile test (right) with indicated gauge length. The loading direction is illustrated with red arrows

Briefly, the stomachs of ten pigs aged 3 months and weighing 80 kg were obtained from a slaughterhouse after animal sacrifice (Horsens, Denmark). The stomach tissue was maintained in phosphate-buffered saline (PBS) saturated gauze at 5 °C until testing. All tests were done within 12 h after removal from the body. Tests were executed at a room temperature of 23 °C. Uniaxial tension and radial compression were done on intact wall samples and separated mucosa and muscularis layers from the antral region (*n* = 36) (Fig. 1). The uniaxial tensile sample length was 45 mm, providing approximately 30 mm of gauge length. The uniaxial tensile sample width was 8.3 ± 0.6 mm. For radial compression, the samples were 29.6 ± 1.9 mm and 30.0 ± 2.3 mm. Intact, mucosa, and muscularis layer thicknesses were 5.7 ± 1.0, 2.2 ± 0.4, and 3.6 ± 1.0 mm, respectively.

Gastric tissue is known for its complex biomechanical behavior, characterized by a combination of elastic responses to external loads (Friis et al. 2022). SOMA devices insert needles at high speeds (above 15 m/s) and the tip is decelerated to zero with the purpose of leaving the tip within the tissue. We have selected intermediate mechanical test conditions. Given these conditions, we did not account for viscoelastic effects as they would have been negligible due to the swift nature of the insertion process. A BOSE ElectroForce® 3200 test bench (225 N load cell (± 0.002 N) and displacement sensor (± 0.001 mm) with WinTest7 software was used for the material tests (Bose Corporation, ElectroForce Systems Group, USA). Before the loading protocol, a pre-load of 1.1 tensile stretch/radial compression was applied. The loading protocol consisted of four cycles of 1.25 tensile stretch/radial compression at a deformation rate of 0.5 mm/s; the first three cycles were preconditioning cycles. The last cycle was used for the analysis of material properties.

### AFM indentation

Five pig stomachs were procured from a slaughterhouse (Berlin, Germany). Experiments were done at the latest 12 h after animal sacrifice. The stomach tissue was kept at 5 °C in PBS-saturated gauze until testing. Tests were executed at a room temperature of 23 °C. Four intact wall samples of approximately 20 × 20 mm from each stomach were cut from the antral region (*n* = 20). The thickness of the intact samples was 4.4 ± 0.6 mm.

**Fig. 2:**
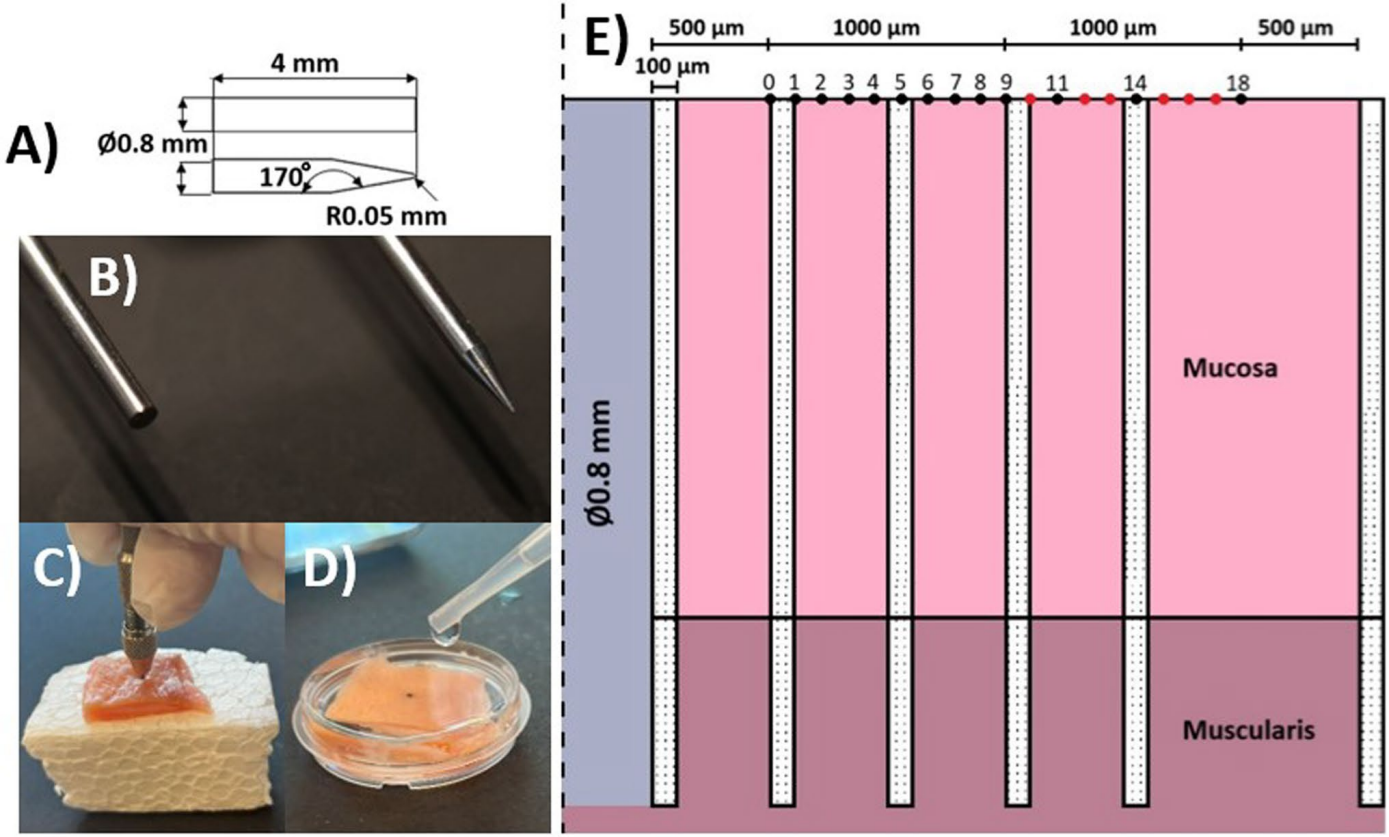
Needle insertion into porcine gastric tissue samples. **A** 2D drawing of the two needles with measures (mm). **B** Close-up image of the two stainless steel needles used for the experimental setup. To the left is the blunt needle, and to the right is the sharp needle with a R0.05 mm tip. **C** A hand tool with the needle is placed to insert the needle manually into the gastric sample. The sample is placed on a polystyrene foam while the needle is gently inserted with the hand tool. **D** Illustration of how the sample is mounted with a needle tip (black dot) into a dish and submerged in PBS. **E** Illustration of the AFM indentation points (0–18), in total, 13 points dispersed, with the first point placed 500 µm away from the needle. The first ten points (#0–9) were divided in a 1000 µm range from the first point to the ninth point. The last three points (#11, 14, and 18) were divided in a 1000 µm range, with an increased distance between each point. The red points just illustrate the distance between the points, as such, they were not analyzed. The YAP-1 areas analyzed are marked with granulated background. Each area had a width of 100 µm. In total, six areas were investigated: next to the needle hole, 500, 1000, 1500, 2000, and 3000 µm away. Two different analyses were made: one with a depth corresponding to the needle hole depth and one corresponding to the mucosa layer thickness. Note that the illustration is cut, not illustrating the whole sample thickness. Furthermore, the illustration is axis-symmetric to the left. Further, note that the AFM test-setup and the YAP-1 test-setup are not executed on the same sample. The inclusion of both test-setups in insert **E** is merely for illustrative purposes

Solid Ø0.8 mm stainless steel needles of 4 mm length with a blunt or R0.05 mm tip (Fig. 2A, B) were manually inserted in the tissue samples (*n* = 10 per needle tip). For this purpose, the needle was attached to a hand tool and gently pressed into the tissue sample placed on expanded polystyrene, so that the needle top was level with the tissue surface (Fig. 2C). Hereafter, the sample was superglued into a plate and submerged in PBS (Fig. 2D).

A JPK02111 CellHesion 200® System with a motorized precision stage and software v.8.0.82 was used for the AFM indentations (JPK Instruments, Bruker Nano GmbH©, Germany). The purpose of the indentations was to determine the elasticity of the tissue samples on a micromechanical level. For this purpose, a spherical indenter was used, as recommended, to reduce the risk of perforation of the sample as well as to reduce pressure applied to the tested area. Furthermore, it was recommended to use a cantilever with a similar stiffness to the sample (Friis et al. 2023). As such, indentations were done with a pre-calibrated rectangular cantilever equipped with a 10 μm spherical tip (SAA-SPH-10 μm, JPK Instruments, Bruker Nano GmbH©, Germany). The cantilever’s spring constant was 0.25 N/m (i.e., compatible with soft samples with moduli from sub-kPa to 100 kPa).

The set point was 6 nN, corresponding to approximately 27–30 nm indentation in the gastric samples. The Z-length and Z-speed were 30 μm and 3 μm/s.

The indentation protocol consisted of 13 indentation points, as illustrated in Fig. 2E. The first point was placed 500 μm from the needle edge to avoid contact between the cantilever and the needle. Ten indentation points (#0–9) were distributed over a distance of 1000 μm (from point 0 to point 9). Three further indentation points (#11, 14, and 18) were distributed over an additional 1000 μm range with an increasing distance between the points. The indentation sequence was randomized using MATLAB’s built-in function to randomize sequences. At each indentation point, 10 indentation cycles were recorded for statistical purposes.

### Biochemical cellular mapping with YAP-1

A preliminary biochemical, cellular mapping experiment was conducted with one pig stomach procured from the animal facility of the Charité (Berlin, Germany). The laboratory pig was used to guarantee the shortest period possible from animal sacrifice to the conduct of the experiment. Samples were prepared and fixed within one hour of termination. From the stomach, 12 intact wall samples of approximately 20 × 20 mm were cut from the antral region (*n* = 12). The thickness of the samples was 7.3 ± 1.0 mm.

Identical needles used for AFM indentation were inserted in the samples (i.e., a blunt needle and a sharp needle, Fig. 2A, B). The 12 samples were divided into three groups, with either a blunt needle inserted (*n* = 4), a sharp needle inserted (*n* = 4), or no needle inserted, i.e. control (*n* = 4). The needle insertion was done as described in Sect. 2.2. After insertion, the samples had a rest period of 30–60 min in PBS pH 7.4 to let the YAP-1 response kick in Franklin et al. (2020). The needles remained in the tissue samples, which were then submerged in Paraformaldehyde (PFA) 4% for 48 h to preserve the tissue samples. After the fixation period, the PFA was replaced with 70% ethanol to dehydrate the samples. Hereafter, samples were embedded in a paraffin wax block and sliced for histological purposes and 8 µm thick slides prepared (Department of Pathology and Imaging, Novo Nordisk A/S, protocol available on request).

To detect the cellular impact of needle insertions, the ISH protocol described below was used to measure the expression of the YAP-1 mRNA molecules in the cells. An automated HCR™ RNA-FISH Technology from Molecular Instruments® (Molecular Instruments, Inc., USA) was used on a Leica Bond RX auto-stainer.

Slides were washed in a 1:10 dilution (Leica Biosystems, AR9590), deparaffinized (Leica Biosystems, AR9222), pretreated with BOND Epitope Retrieval Solution 1 and 2 (Leica Biosystems, AR9962 and AR9640) and protease (ACD, 322750) followed by probe hybridization. Probes were detected using the RNAscope LS Multiplex Reagent Kit (ACD, 322750), and bond polymer refine (red) detections (Leica Biosystems, DS9390, and DS9800) were applied. Furthermore, green and blue chromogens were used in the staining process (Leica Biosystems, DC9913 and DC9896). Slides were counterstained with Hematoxylin and bluing (Leica Biosystems, AR9915) (Department of Pathology and Imaging, Novo Nordisk A/S). Results from this protocol include images where YAP-1 mRNA is detected with red.

Measurements were taken at the following distances from the needle hole: 0, 500, 1000, 1500, 2000, and 3000 μm (Fig. 2E). The analysis at each distance used a region of interest (ROI) being 0.1 × 5.8 ± 1.4 mm, encompassing distances of 0 to 3000 μm away from the insertion site. Furthermore, the mucosa layer was analyzed using the same approach. Specifically, YAP-1-stained slides were scanned using a Hamamatsu Nanozoomer S60 slide scanner at a magnification of 40×. The scanned slides were subjected to analysis using the Visiopharm Integrator System (Visiopharm, Denmark). Image analysis involved the application of a threshold analysis in the red channel, with a preprocessing filter of Chromaticity Red, Mean (0.4) using Visiopharm Integrator System (VIS). Within the defined ROIs, the area of the YAP-1 ISH signal was quantified. Data were reported as density (YAP-1 signal in the total area measured) per ROI. The muscularis data were found by subtracting the first analysis and the mucosa analysis from each other (Fig. 2E).

### Data and statistical analyses of experimental tests

Data analysis and statistical work were done using MATLAB version 9.9, R2020b (MathWorks, Inc., Natick, Massachusetts, USA), Microsoft Excel 2016 (Microsoft Corporation), and GraphPad Prism 10.0.2 (GraphPad Software, Boston, Massachusetts, USA). Data were expressed as mean and standard deviation (SD) and/or with quartiles (Q1 being the 25th percentile, Q2 being the median, and Q3 being the 75th percentile). The specific data and statistical analysis for each experiment follow in the next sections.

#### Work curves from mechanical tests

Raw data from tensile and compressive tests were extracted from Friis et al. (2023). Here work curves are described by functions for the stress development as function of strain as shown in supplementary materials, Table S-1. This fitting procedure and statistical treatment is described in Friis et al. (2023).

#### AFM indentation

To determine Young’s moduli, i.e., the stiffness of the tissue in the indented areas, SPM Data Processing Software, version 8.0.85 (JPK Instruments, Bruker Nano GmbH©, Germany) was used. The software employed a Hertz elasticity fit to the data output (height positions for the approach/the vertical tip position and the vertical deflection in the form of spectroscopy curves).

In this study, a spherical tip was used. Thus, the Hertz model was expressed as two equations relating the applied force, *F*, and the indentation, *δ*, into the tissue to the contact area, *a*:

1 $$F=\frac{E}{1-\nu }\cdot \left[\frac{{a}^{2}+{R}^{2}}{2}\cdot \text{ln}\left(\frac{R+a}{R-a}\right)-a\cdot R\right]$$

2 $$\delta =\frac{a}{2}\cdot \text{ln}\left(\frac{R+a}{R-a}\right)$$

here *E* is the elastic modulus, *R* is the radius of the sphere, and *ν* is Poisson’s ratio. From this, Young’s moduli were derived by a fitting procedure. For soft biological materials, *ν* is often set to 0.5, recognizing the sample as an incompressible material. This was also assumed in this study.

All data sets were assessed for normal distribution using the Anderson–Darling normality test. Data were considered normally distributed if *p* > 0.05 (see Supplementary Materials, Table S-1 for specific *p*-values).

A linear regression fit to the data was assessed in terms of confidence intervals and the goodness of fit (*R*^2^). Covariance (ANCOVA) was analyzed to test whether the linear regression slopes and intercepts for the blunt and sharp needle geometries differed. *p* < 0.05 were considered significant.

Furthermore, it was tested if the stiffness measured at indentation point 0 significantly differed from the stiffness measured at indentation point 18 using an unpaired *t*-test assuming unequal variance for normal distributed data. For nonparametric data, the Wilcoxon rank sum test was used. *p* < 0.05 indicates a difference between the stiffness measured in the two indentation points.

#### Biochemical cellular mapping with YAP-1

The YAP-1 mRNA expressions in each of the five areas were determined from the auto-stained images. The expression was calculated as the density/fraction (in permille, ‰) of tissue area versus the area in the same ROI that was red from the nuclear staining.

Due to the small number of measures from one pig only, it was chosen to plot all data points for each analyzed area instead of mean or median.

The same statistical approach as for the AFM indentation was used to analyze the distance-dependent YAP-1 behavior: A linear regression analysis, evaluated in terms of confidence intervals and by goodness of fit (*R*^2^), analysis of covariance (ANCOVA), and Wilcoxon rank sum test testing if the YAP-1 expression 500 μm away from the needle hole was significantly different from measures taken 2000 μm away from the needle hole. *p* < 0.05 were considered significant.

### Numerical simulations

Computer simulations applied to acquire solutions to the elastic problem, as outlined in Fig. 4, follow the theory from Reddy (2013). In short, the finite element method as implemented in COMSOL Multiphysics® version 6.3 (COMSOL AB, Stockholm, Sweden) is used to solve the following sets of coupled governing equations (Eqs. 3, 4, and 5).

The strain–displacement equation expressed by the Green–Lagrange strain tensor, *E*, which accounts for large relative displacements as described by *u*:

3 $$E=\frac{1}{2}\left[\nabla u+{\left(\nabla u\right)}^{T}+\left(\nabla u\right){\left(\nabla u\right)}^{T}\right]$$

where *T* denotes the transpose operation.

The second governing equation is the principle of conservation of linear momentum, also known as Newton’s second law of motion, that balances acceleration and mass to forces expressed as the gradient of stresses within the tissue:

4 $${\nabla }_{0}\bullet {\left(S\bullet F\right)}^{T}+{\rho }_{0}f={\rho }_{0}\frac{{\partial }^{2}u}{\partial {t}^{2}}$$

where $S$ represents the second Piola–Kirchhoff stress tensor, $F$ is the deformation gradient, ρ_0_ is density, f represents the body force per unit mass applied to each point, and $\frac{{\partial }^{2}u}{\partial {t}^{2}}$ is the acceleration. The first Piola–Kirchhoff stress tensor $P=S\bullet F$.

Thirdly, constitutive equations express the behavior of tissues by stating a correlation between stresses and strains that are experimentally verified. It was previously found that the hyperelastic Fung model captures stomach behavior very well (Friis et al. 2023; Patel et al. 2022). This study applies the energy function for the generalized Fung anisotropic hyperelastic model (Patel et al. 2022):

5 $$W=\frac{c}{2}\left[\text{exp}\left(Q\right)-1\right]+\frac{\kappa }{2}{\left(J-1\right)}^{2}-\text{p }(\text{J}-1)$$

where $Q=E:b:E$, $E$ is the Green–Lagrange strain tensor and $b$ a fourth-order tensor of dimensionless material parameters, $c$ is a stress like material parameter, and $\kappa$ is the bulk modulus, penalizing changes in volume. *J* is the determinant of the deformation gradient expressing volumetric strain changes. *J* = 1 will indicate no volume change, as the case for incompressible media. $p$ is the hydrostatic pressure.

The Cauchy stress for the generalized Fung model is:

6 $$\sigma =\frac{1}{J}F\frac{\partial W}{\partial E}{F}^{T}+\frac{\partial W}{\partial J}I$$

where *F* is the deformation gradient tensor and *I* is the identity tensor.

#### Material models and model calibration

To establish the Fung constitutive model, numerical simulations of the circumferential and longitudinal tension and radial compression setups were made to resemble experiments previously conducted (see Sect. 2.1). Then, the Levenberg–Marquardt algorithm as implemented in Comsol was used to solve the optimization problem that compares the experimental results and a finite element model of the experiment. A detailed description of its implementation is found at Comsol’s application gallery (2025). It updates the material parameters to minimize the difference between the two. It can be formulated mathematically as a nonlinear least-squares minimization problem:

7 $${{\varvec{q}}}_{\text{opt}}={\text{argmin}}_{{\varvec{q}}}\left(\sum_{n=1}^{N}{Q}_{n}\right)$$

with

8 $${Q}_{n}=\frac{1}{2}\sum_{m=1}^{M}\left({P}_{n}\left({\lambda }_{m},{\varvec{q}}\right)-{\widehat{P}}_{nm}\right)$$

where ${\varvec{q}}$ is the vector of material parameters wanted to be estimated (see Eqs. 5 and 9), $N$ is the number of experiments, ${M}_{n}$ is the number of data points per experiment, $\text{and } {\widehat{P}}_{nm}$ is the *m*th data point of experiment *n*. These data are extracted from Table S-1. ${P}_{n}\left({\lambda }_{m},{\varvec{q}}\right)$ denotes the corresponding model prediction given the experimental parameter ${\lambda }_{m}$ following (COMSOL 2025; Sferrazza et al. 2019).

In these computations, incompressibility is assumed. Furthermore, due to the availability of experimental data it is assumed that anisotropic properties of the tissue can be captured by a limited number of b coefficients reducing the expression for Q to:

9 $$Q={b}_{rrrr}{E}_{rr}^{2}+{b}_{zzzz}{E}_{zz}^{2}+{b}_{\theta \theta \theta \theta }{E}_{\theta \theta }^{2}$$

Following previous implementations, a linear constitutive model was used to describe cellular behavior (Katzengold et al. 2015); however, the current study applies a more recent understanding of how cells regulate their cytoskeletal stiffness to their surrounding matrix (Janmey et al. 2020). The nucleus is the largest and stiffest organelle in a cell and was assumed to be twice the stiffness of the cytoplasm following the reference (Luo et al. 2016).

A cell's expression of YAP-1 is sensitive to its local stress field. Based on the work by Al-Nuaimi et al. (2024) and Venturini et al. (2020), we hypothesized that YAP-1 expression correlated to a hydrostatic stress state formulated as:

10 $${\sigma }_{h}=\frac{1}{3}\text{trace}(\sigma )$$

#### Numerical needle insertion

**Fig. 3:**
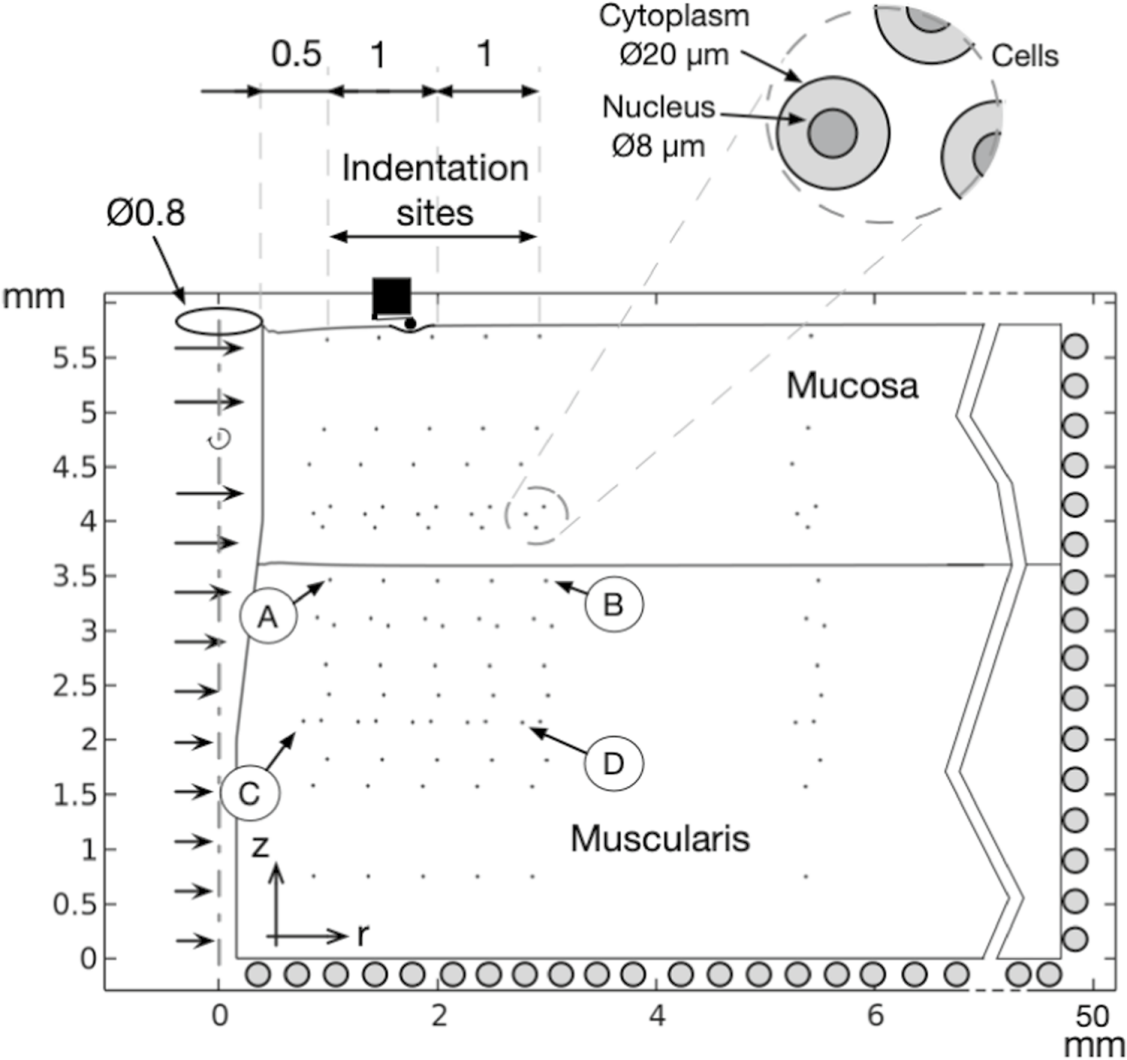
Geometrical description of tissue layers, its deformation when subjected to a Ø0.8 mm needle insertion (here, the sharp needle shown), and the location of individual cells. Rotational symmetry around the *z*-axis. A radial prescribed deformation is assigned to denote the presence of a needle. At the bottom and the far-right, tissue can move tangentially to the *r*- and *z*-axis. The A, B, C, and D markers indicate cells included in plots of hydrostatic stresses and curvature of the nuclear pore

The consequences of needle insertion were numerically investigated by solving the elastic problem of the geometry shown in Fig. 3. The model consisted of the tissue regarded as two separate layers, namely mucosa and muscularis, with a thickness of 2.2 and 3.6 mm layer, see Table 1 in Friis et al. (2023). It included 96 biological cells with cytoplasmic and nuclear domains. The large length-scale difference between cellular organelles and tissue, together with the number of cells included, will lead to a large 3D computational problem. To reach a more computational efficient problem that allow inclusion of a higher number of single cells, the setup was simplified to be 2D axisymmetric. The axis of rotation is on the left side in Fig. 3. The number of cells is chosen to provide a spatial distribution sufficient to capture cellular variations in stress, while also limiting the number of mesh elements required to achieve computational efficient and mesh-independent solutions.

**Table 1:** Material properties of porcine gastric tissue (mucosa and muscularis layers) were extracted from Friis et al. (2023) and incorporated in the anisotropic, hyperelastic Fung model

Tissue layers	*c* [Pa]	${b}_{rrrr}$	${b}_{zzzz}$	${b}_{\theta \theta \theta \theta }$
Mucosa	101.46	20.34	0.39	17.49
Muscularis	12.20	20.45	11.24	11.87

The material parameter, *c*, indicates the stiffness of the samples. The material parameter, *b*, arise from Eq. 9 and encode material anisotropy. The subscripts indicate the coordinate-system orientation (see Fig. 3)

Within the mucosa and muscularis layers, at a radial distance of 900 μm and at a height of 3.5 mm, 16 cells were located. They were then randomly moved ± 100 µm along the *r*-axis and ± 2 mm along the *z*-axis as determined by a randomization function. This pattern was repeated in the radial direction at 1400, 1900, 2400, 2900, and 5400 µm. Cell dimensions follow own histological observations and the literature (Holzer et al. 2024). Based on the choice of cellular modeling described in Sect. 2.5.1, the cytoplasmic stiffness was selected to 0.5 kPa and thus a nuclear stiffness of 1 kPa.

Blunt and sharp cylindrical needles of Ø0.8 mm are considered (see Fig. 2A, B). To simulate the presence of a blunt needle to a depth of 2 mm, a straight and radial movement was imposed at the left edge, repositioning it to *r* = 400 µm. To simulate the presence of a sharp needle, the same edge was moved in the radial direction, reflecting the tip geometry as shown in Fig. 3.

To address convergence issues arising from topological changes and mesh distortions due to significant radial movements, an initial hole of 160 µm for needle insertions was necessary.

To extract indentation information, 22 vertices were located at the tissue surface from a radius of 900 µm and for every 1000 µm, consistent with the placement of the indentation points in the experimental AFM indentation setup (Sect. 2.2).

The radial width of the tissue was 50 mm, large enough to ensure that the needle disturbance to the strain field was leveled out. At the lower and far-right surface of the tissue, the movement was constrained to be in the plane of the surface.

A triangular mesh was used to discretize the geometry. Care was taken to mesh the nucleus and cytoplasm by using a mesh size of 0.5 µm and then gradually increasing element size into the tissue to a maximum size of 100 µm. Quadratic Lagrange polynomials were used as shape functions. The complete finite element problem was solved on a Hewlett Packard Z2 Tower G9 Workstation with an Intel i9-13900K chip and 128 Gb of memory. It consists of 1.097.392 triangular elements, having 2.781.595 degrees of freedom. The solution time is 2.9 h, establishing incremental solutions using a peak memory allocation of 27 Gb.

#### Validation of numerical model

To validate the mechanical tissue and cell response in the simulations, AFM indentation around the needle insertion site was numerically conducted. This method reveals the local indentation stiffness at the tissue surface at sites, as shown in Fig. 2E. This experimental AFM measured stiffness is compared against the numerical stiffness at similar sites calculated as:

11 $${C}_{zz}=\frac{{\sigma }_{zz}}{{E}_{zz}}$$

## Results

### Material model

The constitutive equation that models the anisotropic and hyperelastic behavior of the stomach tissue, is based on the experimental mechanical model from Friis et al. (2023). From the calibration procedure, fitting the experimental stress–strain data with the Fung model (Eq. 9) provided the parameters listed in Table 1.

### AFM indentation (experimental and numerical)

**Fig. 4:**
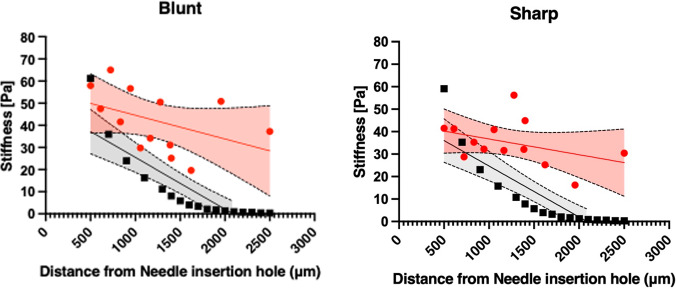
AFM plots. The red points illustrate the experimental mean values. The red line is the regression line, and the red area illustrates the CI bands (mean, SD, and quartile measures can be found in Supplementary Materials, Table S-2 and Table S-3). The black data points are generated from the simulation of the AFM setup. The black line is the regressions line, and the grey area illustrates the CI bands. The left plot is for the blunt needle, and the right plot is for the sharp needle

For both needle geometries, the stiffness measured from AFM indentations decreased with the distance to the needle insertion point (Fig. 4).

The numerical model was tested by executing the AFM indentation as a simulation (Fig. 4, black data) and comparing it against experimentally measured AFM data (Fig. 4, red data).

The experimental and numerical data for both the blunt and sharp needles showed a similar downward trend, demonstrating that the FEA model captures the primary behavior of the system, i.e., a consistent decrease in stiffness with increasing distance from the insertion site. The regression lines indicated that stiffness in the blunt needle samples decreased from approximately 55–30 Pa experimentally and from approximately 44–12 Pa numerically. The sharp needle decreased from 43 to 25 Pa experimentally, and the numerical stiffness from 49 to 7 Pa.

For the blunt needle, the numerical trend line had a steeper slope than the experimental data, indicating that the model predicts a quicker decrease in stiffness than observed experimentally. The numerical data for the sharp needle show an even steeper slope than the experimental data. The experimental and numerical data in the blunt needle plot align well at shorter distances (up to about 1000 μm). Beyond this range, the numerical data show a steeper decline in stiffness. For the sharp needle, initially, the numerical model follows experimental data closely but diverges at longer distances, with the numerical model showing a steeper reduction in stiffness.

From experimental data, statistical analyses indicated that the differences in stiffness, measured 500 µm away from the needle insertion site (point 0) and 2500 µm away from the needle site (point 18), were significant for both needle geometries (*p* < 0.05; see Supplementary Materials, Table S-4 for specific *p* values).

Differences in slope and intercept from the linear regressions were insignificant between the blunt and sharp needle (experimental measures) (*p* > 0.05; see Supplementary Materials, Table S-5 for specific p-values).

### Cellular mapping with FEA and experimental YAP-1

**Fig. 5:**
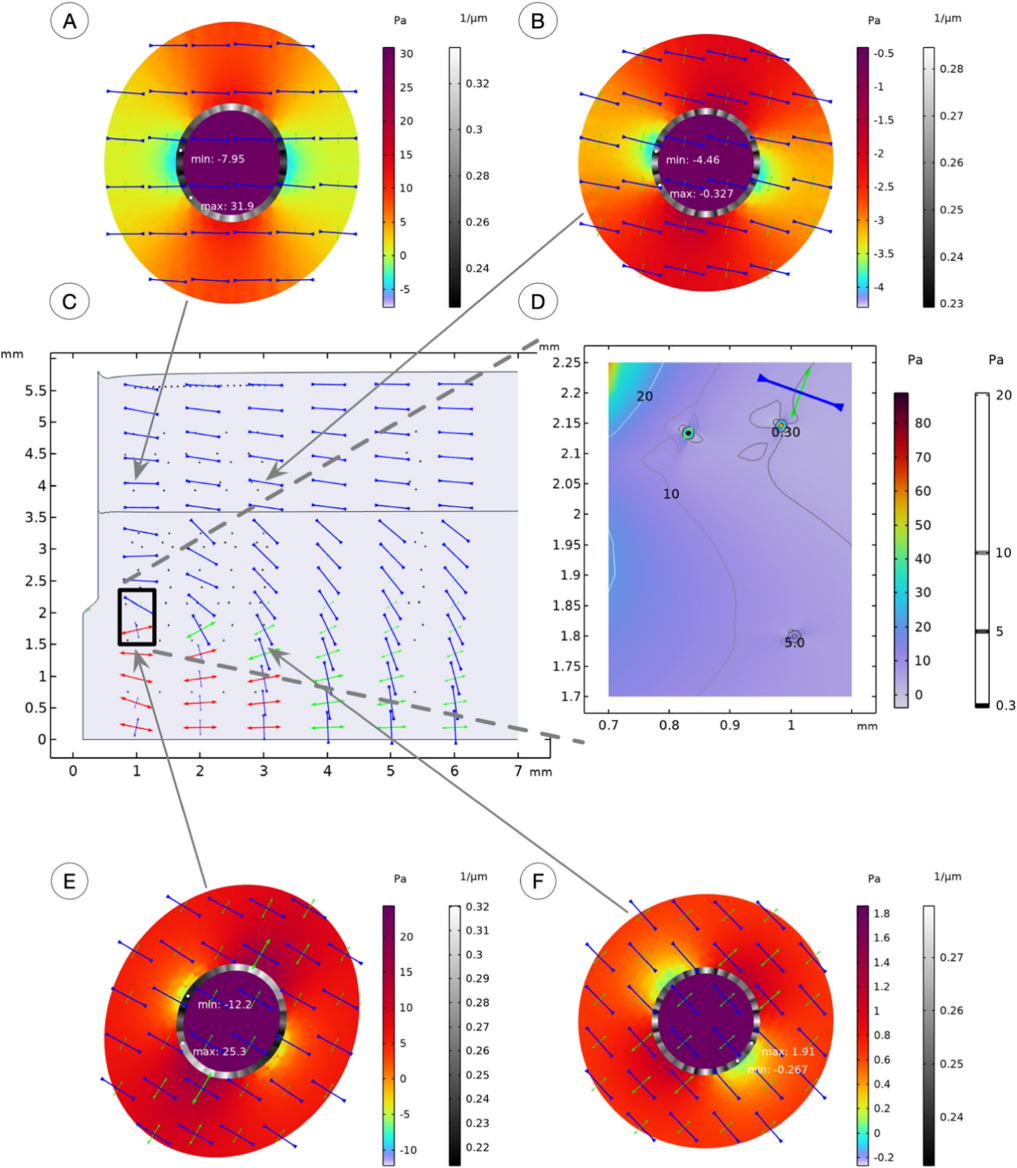
Stress fields at tissue (**C**, **D**) and single cell (**A**, **B**, **E**, **F**) length scales caused by a blunt needle insertion. Depicted crosses show the orientation of principal stress directions. The principal stresses ${\upsigma }_{1}$, ${\upsigma }_{2}$, and ${\upsigma }_{3}$ are ordered and plotted so that ${\upsigma }_{1}$ (red) > ${\upsigma }_{2}$ (green) > ${\upsigma }_{3}$ (blue). The arrows' direction determines if the stress state is tensile or compressive at the location of the cross. Color plots correspond to the level of hydrostatic stress, and greyscale plots correspond to the curvature of the nuclear pore membrane

**Fig. 6:**
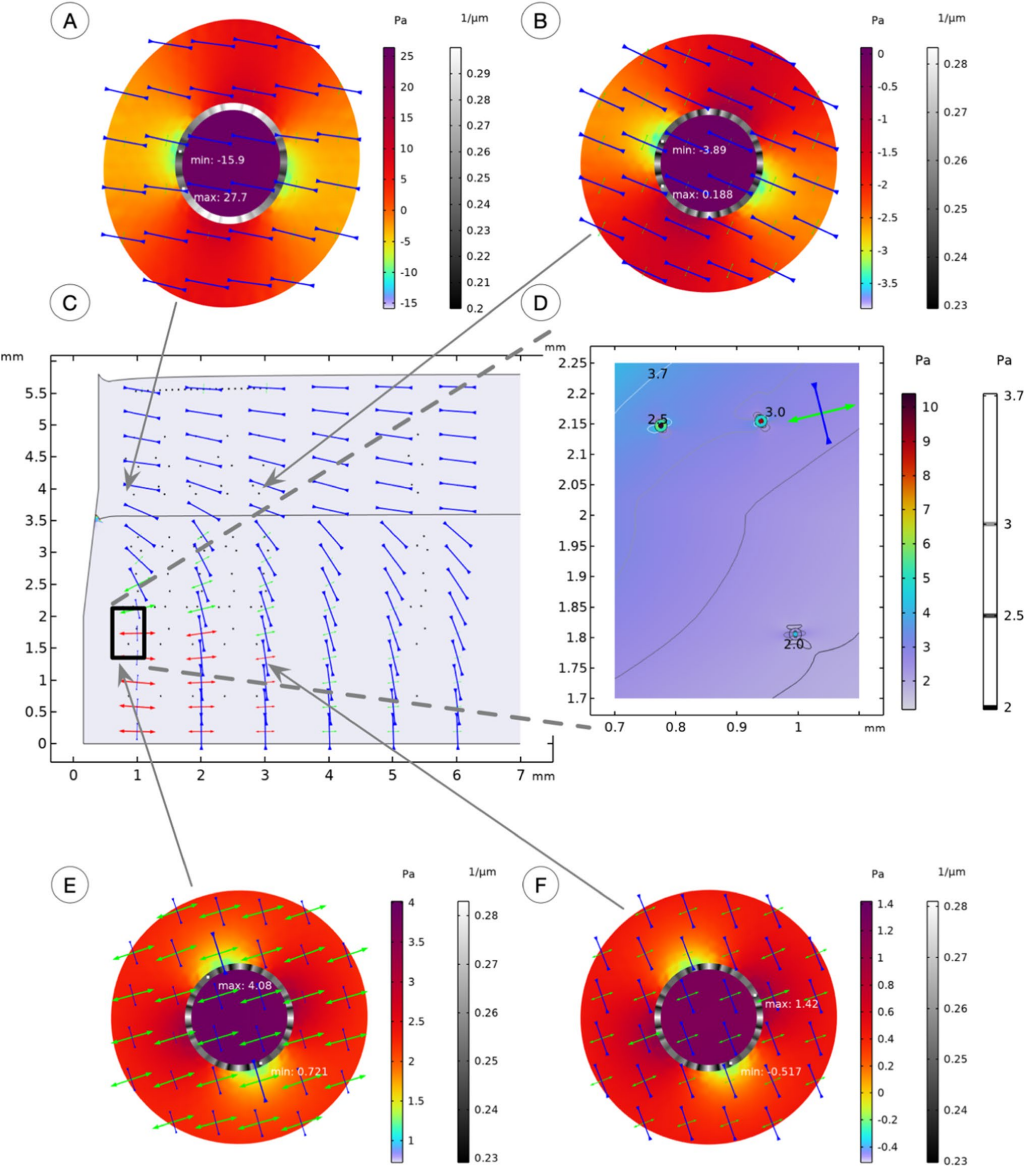
Stress fields at tissue (**C**, **D**) and single cell (**A**, **B**, **E**, **F**) length scales arise from a sharp needle design. Depicted crosses show the orientation of principal stress directions. The principal stresses ${\upsigma }_{1}$, ${\upsigma }_{2}$, and ${\upsigma }_{3}$ are ordered and plotted so that ${\upsigma }_{1}$ (red) > ${\upsigma }_{2}$ (green) > ${\upsigma }_{3}$ (blue). The direction of the arrows determines if the state of stress is tensile or compressive at the location of the cross. Color plots correspond to the level of hydrostatic stress, and greyscale plots correspond to the curvature of the nuclear pore membrane

Plots of the stress field in the overall tissue and localized to individual cells for the blunt (Fig. 5) and sharp (Fig. 6) needles were extracted. Arrows show the magnitude and principal directions of the stress field within the tissue in insert C. These reveal how cells located in the mucosa were exposed to a compressive state orientated along the surface. On the contrary, cells located in the muscularis next to the needle tip realized a rotation in the highest stress together with a shift from a predominantly compressive state to a tensile state of stress. The complex stress field created by the needle is influenced by two main factors: the prescribed deformation of the tissue caused by the needle's presence and the tissue's anisotropic properties. This stress field tends to stabilize in the radial direction as you move away from the needle.

The local stress field to individual cells is shown in insert D. In this approach, cells are situated in a homogenized ECM, and its anisotropic properties induced a butterfly-like variation of the 1st principal stresses that emerge from the needle insertion. This implied that the cell membrane realized a simultaneous state of compression and a state of tension on two opposite sides of the cells.

As a balance of forces is maintained over the cell membrane, this external stimulus was reflected by the intracellular stress field, as shown for four individual cells in inserts A, B, E, and F. The intracellular plots show the hydrostatic stress exerted onto the cytoskeleton and carried to the nucleus. As the mechanical properties of the homogenized cytoskeleton are different from the properties of the nuclear condensate, different stress levels in the two domains were observed. Most significant variations are seen in the cells close to the needle. Their cytoplasmic compartments experience both hydrostatic compression and tension, depending on the position within the cytoskeleton. The arrows within each cell show the direction of the largest 1st principal stresses.

Nuclear pore membranes were deformed and curved because of the cells' stress fields. The thickness of this membrane has been enhanced to improve visualization. In its reference configuration, the membrane curvature was 0.25 1/µm and departed from there to vary from 0.17 to 0.42 1/µm in the most oval case, next to the blunt needle tip.

The blunt needle gave rise to a higher localized stress field near the needle tip and variations to the principal directions in the far field 5 mm from the needle tip in the muscularis tissue layer. In front of the needle tip, the blunt design gave rise to a compressive state in the z-axis direction (along the needle) and a tensile state in the radial direction. This is contrary to the sharp needle that introduced a tensile state in the z-axis and a compressive state in the radial direction. The directional rotation of the principal stress directions was gradually introduced by a sharp needle and more abruptly by a blunt needle (see Figs. 5 and 6, just above the black square marking insert D).

**Fig. 7:**
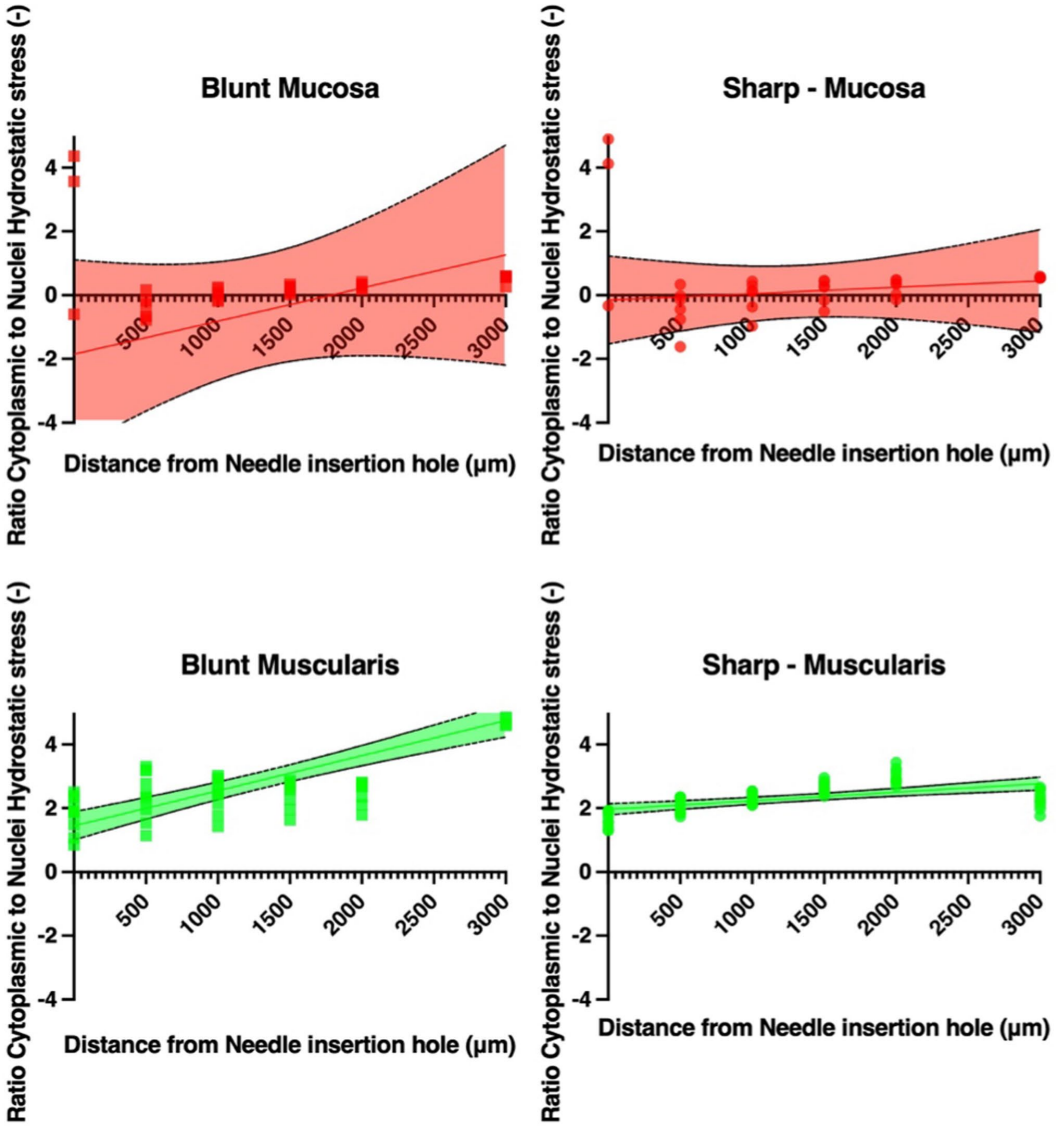
Nuclei to cytoplasmic hydrostatic stress ratio as a function of the distance from the needle insertion. The plots are for blunt needles (left) and R0.05mm needles (right) for mucosa, and muscularis. Data points are the mean values of all data points for each simulated measurement area. On the plots is a linear regression, and CI bands are included. Be aware of the different *y*-axes

From these simulations, ratios of nuclei to cytoplasmic hydrostatic stresses are plotted as a function of the distance to the needle insertion hole in Fig. 7. These values are extracted as average values of hydrostatic stresses, ${\sigma }_{h}$ [Pa], in the domains of single cells distributed in the mucosa and muscularis layer. The plots indicate a slightly increasing trend as a function of the distance to the needle hole for the intact and mucosa layers. The sharp needle ratio was smaller than for the blunt needle.

**Fig. 8:**
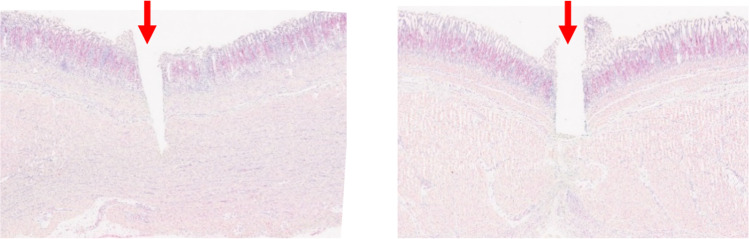
Histological images of YAP-1 staining (red). The left image illustrates a sharp needle insertion, and the right image represents a sample with a blunt needle insertion. Red arrows mark the needle insertion points. For high-resolution images, see Supplementary Materials, Figure S-1 and Figure S-2

The staining of YAP-1 expression provided images, as shown in Fig. 8 for one sample with a blunt needle and one with a sharp needle. For high-resolution images, see Supplemental Material, Figures S-1 and S-2.

**Fig. 9:**
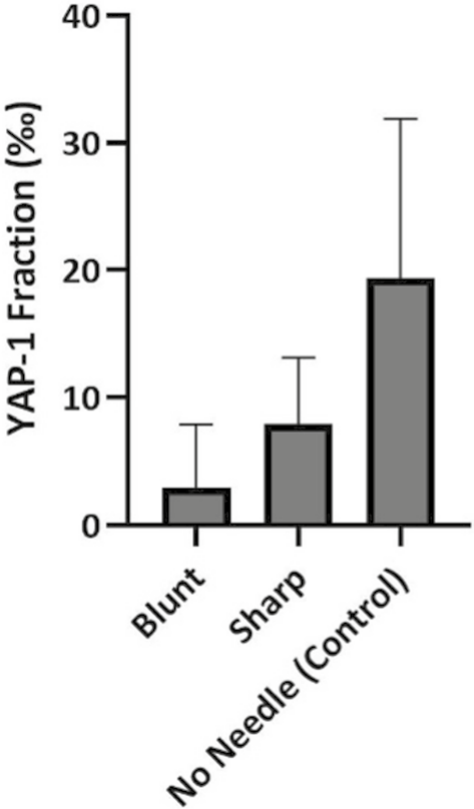
YAP-1 fraction (‰) measures inside blunt and sharp needle holes, as well as a control measure with no needles inserted. The plot shows the median value and outer ranges

The fraction in permille (‰) is plotted in Fig. 9 for intact samples with measures inside the blunt and sharp needle holes, as well as a control measure with no needles inserted. The plot shows the median value and outer ranges. A qualitative analysis of the plot indicates that the control sample had a higher fraction value of YAP-1 than the samples injected with needles.

**Fig. 10:**
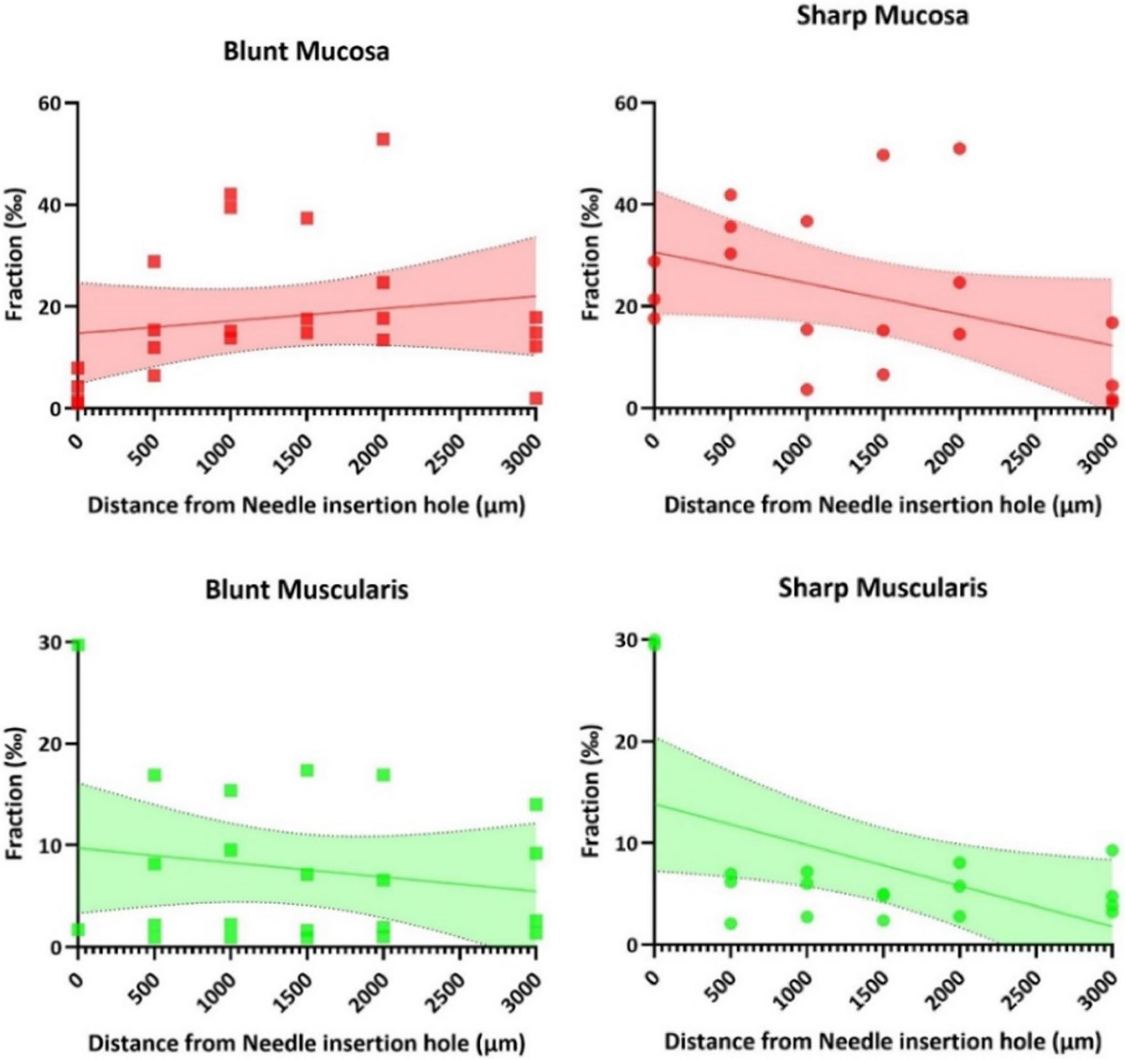
YAP-1 fraction (‰) as a function of the distance to the needle insertion. The plots are for blunt needle (left) and R0.05 mm needle (right) for mucosa, and muscularis. All data points are included for each measurement area. On the plots are linear regression and CI bands included. Be aware of the different *y*-axes

The fraction in permille (‰) is plotted in Fig. 10 for the mucosa, and muscularis layers as a function of the distance from the needle hole. The plots indicate an increasing trend for the blunt needle in mucosa and a decreasing trend in muscularis, whereas the sharp needle implies a decreasing trend as a function of the distance to the needle in both layers. The fractions obtained in the area 500 µm and 2000 µm away from the needle hole did not significantly differ (*p* < 0.05; See Supplementary Materials, Table S-7). ANCOVA from the linear regression indicated no significant differences in intercept or slope for the two needle geometries (*p* > 0.05; see Supplementary Materials, Table S-8).

## Discussions

Oral drug devices targeting the GI wall are promising for macromolecular delivery, yet their mechanical and cellular impacts remain underexplored. This study presents a novel mechanobiological framework that combines experimental and numerical methods to assess ex vivo gastric needle insertion. This study is unique in its integration of tissue mechanics and cellular responses. We developed and validated a computational model using tensile and radial compression data, confirmed with AFM, and examined cellular impacts numerically and via YAP-1 mRNA expression, offering new insights into the biomechanical and cellular effects of different needle geometries.

### Atomic force microscopy indentation

AFM has been widely used to study the micromechanical properties of soft tissues, making it a valid method for investigating the changes in stiffness in gastric tissue samples in the current study. Earlier studies have demonstrated AFM's capability to capture subtle mechanical variations, reinforcing its reliability. For example, Lin et al. (2020) examined the micromechanical properties of esophageal mucosa using AFM, providing insight into soft tissue stiffness (Lin et al. 2020). The study highlighted AFM’s sensitivity in capturing tissue properties, supporting its use in this research. Micro-indentation techniques applied to murine articular cartilage revealed the influence of tissue heterogeneity on stiffness, comparable to the observations in the current study (Arnold et al. 2023). Also, investigations on gastric cancerous tissue have shown notable variability in stiffness due to pathological tissues, aligning with findings by Taheri and Farahani of localized stiffness variation (Taheri and Sousanabadi 2024). This supports the idea that AFM can effectively capture stiffness differences, which reflect underlying structures in normal and pathological tissues or, as in our case, an inserted needle. A comprehensive review has highlighted AFM’s role in disease-related studies, emphasizing its strength in detecting minute changes in tissue stiffness that signal pathology. The distance-dependent behavior in the current results aligns with these observations, demonstrating AFM’s efficacy in assessing mechanical properties in healthy and diseased tissues (Liu et al. 2024).

Our hypothesis proposed that radial displacement around the needle would generate compressive radial stress, accompanied by a more complex triaxial stress state due to the anisotropic properties of the tissue. This stress state is associated with an equally complex strain field. The observed change in stiffness measured by AFM may result from local stress components aligned with the direction of the AFM measurements or from tissue densification. The analysis for both needle geometries indicated explicit distance-dependent elastic behavior, as evidenced by the significant reduction in stiffness with increasing distance from the needle tip. These trends confirm the initial hypothesis that the presence of the needle induces localized compression in the surrounding tissue, leading to altered mechanical properties. The significant differences in stiffness between the initial measurement point and the final measurement point for both needle types highlight the impact of needle insertion on tissue mechanics. The observation that the blunt needle exhibited a steeper linear regression than the sharp needle suggests that the blunt needle creates a more pronounced stiffness gradient within the tissue. This indicates that the blunt needle engages a larger tissue area. The difference in the stiffness profiles could have clinical implications, particularly in procedures requiring a gentler approach, as the sharp needle's characteristics might result in less traumatic interactions with surrounding tissues. Despite the differences in stiffness values and the steeper regression for the blunt needle, it is noteworthy that the linear regression coefficients between the needle geometries did not differ significantly (*p* > 0.05). This implies that while the blunt needle leads to more significant stiffness changes, the overall stiffness behavior across the tested distance range is comparable between the two geometries. This finding may suggest that both needle types ultimately affect tissue mechanics similarly in the context of elastic behavior, albeit through different mechanisms. Moving forward, exploring the underlying mechanisms contributing to the differences in stiffness profiles between the needle geometries would be beneficial. Investigating additional parameters, such as the tissue's viscoelastic properties and the indentation rate, will provide a more nuanced understanding of tissue response to needle insertion.

### Numerical model

The numerical model's validation was assessed by comparing the experimental AFM indentation results with simulated outcomes for both needle geometries. The model successfully captured the observed distance-dependent stiffness behavior, demonstrating a decrease in stiffness with increasing distance from the needle insertion point. This agreement between experimental data and numerical predictions supports the model's accuracy in simulating tissue mechanics under localized compression. Furthermore, it indicates that the simplifications inherent in the model are justified for broader predictive use.

While the model reflected the general trend seen in the experiments, some deviations were noted, particularly in capturing absolute stiffness values at specific points. These differences could be attributed to model simplifications, such as assumptions regarding tissue homogeneity or boundary conditions. Nonetheless, the consistent alignment in the rate of stiffness change between experimental and numerical results reinforces the model's robustness.

The gap between experimental and numerical data was more pronounced for the blunt needle than the sharp needle, indicating potential differences in how well the numerical model represents reality in these cases.

Given the viscoelastic nature of biological tissues, our model can be extended to include time-dependent solutions; further refinement incorporating detailed tissue heterogeneity, biochemical reaction kinetics, or nonlinear properties will enhance its predictive capability. Overall, the validation outcomes indicate that the current model is a reliable tool for simulating tissue interactions with needle geometries, providing a foundation for future explorations into optimized needle design and minimally invasive techniques.

### Cellular mapping

The mechanical stress fields and cellular regulation of YAP-1, known to be mechanosensitive, were examined to identify their correlations.

The stress field generated by a needle disturbance is complex within our anisotropic, heterogeneous, hyperelastic model, affecting both tissue and single-cell length scales. According to the work of Tajik et al. (Tajik et al. 2016), each individual cell acts as a force sensor, detecting where forces impact its membrane and regulating its gene expression accordingly. The variation of stresses along the cell membranes leads to a complicated cellular response that depends not only on the presence of a force but also on its direction, magnitude, and temporal variation.

Literature provides various interpretations of how cells perceive mechanical signals. There are two distinct mechanisms related to where the signal is sensed. First, if the signal is detected at the cell membrane, it activates pathways that ultimately lead to cellular adaptation (Eroumé et al. 2021). Alternatively, the signal may be transmitted directly to the nucleus, resulting in its reshaping and condensation (Ghagre et al. 2024). This mechanism is influenced by the fact that the elastic wave generated when a needle is inserted propagates through tissue at the speed of sound, also traveling through the load-carrying structures of the cytoplasm (Zheng et al. 2013). Consequently, a mechanical signal can reach the nucleus almost instantaneously, in contrast to a diffusion–reaction-driven signal that emerges from the membrane (Shamir et al. 2016). Our simulations of this elastic problem demonstrate that mechanical signals reach the nucleus instantaneously, leading to observable deformation, such as changes in curvature. Since YAP-1 regulation involves an exchange between the nuclear and cytoplasmic compartments, it is essential to adopt an integrated approach that captures both mechanical and chemical regulations to fully understand this process.

The plots illustrating the ratio of nuclei to cytoplasmic hydrostatic stress in Fig. 7, as well as the YAP-1 fraction in Fig. 10, qualitatively demonstrate a YAP-1 fraction which is opposite in slope of the hydrostatic stress as function of radial distance from the needle. The blunt needle produces ambiguous results to this in the mucosa layer. This discrepancy may suggest that the geometry of the needle affects the YAP-1 response in different ways. However, the YAP-1 results in the current study did show a distance-dependent behavior although not statistically significant. This correlates with the distance-dependent stiffness observed in the AFM measurements.

YAP-1 is a key regulator in mechanotransduction, influencing cellular responses to mechanical cues. It plays an essential role in tissue remodeling and stiffness perception, yet its response to localized mechanical stimuli, such as the needle insertion in the current experiment, was not as pronounced as anticipated. One possible explanation for the absence of a distance-dependent response is that the affected area is much smaller than anticipated, and the distances from the insertion site should have been smaller increments than the ones used in the current study. Furthermore, we measured YAP-1 mRNA coding for YAP-1 protein. It is, to our knowledge, the first time such a study has been executed. Thus, it is unknown if YAP-1 mRNA would at all be regulated in ex vivo non-vascularized tissue.

Beyond YAP-1, additional regulators may be relevant for linking mechanical stimuli to gene expression, potentially offering complementary perspectives in our study. Examples include TAZ (transcriptional coactivator with PDZ-binding motif), a partner in the Hippo pathway that is also mechanically sensitive, and β-Catenin, which responds to cell adhesion and tension, mainly through Wnt signaling pathways. Integrins and Focal Adhesion Kinase (FAK) are similarly promising, as they mediate downstream transcriptional signaling in response to external mechanical stimuli. Future studies exploring the roles of these additional regulators could provide further insights into the complex pathways linking biomechanical signals to transcriptional responses (Totaro et al. 2018; Hong and Guan 2012).

### Limitations

Due to practical constraints, porcine tissue was employed instead of human tissue despite prior evidence of interspecies differences in mechanical properties (Friis et al. 2023). This substitution, while expedient, may limit the model’s direct applicability to human tissue. Future studies must prioritize human tissue where feasible or apply interspecies adjustments based on previously documented mechanical differences. Enhancing the translatability of results to human tissue is critical, especially for applications relevant to human clinical outcomes.

The current study utilized the incompressible generalized Fung model to represent tissue in computational simulations calibrated through uniaxial tensile and radial compression tests. Although this approach enabled us to approximate tissue behavior, some limitations must be noted. The generalized Fung model, which was calibrated using uniaxial tensile and radial compression tests, worked well for approximating how the tissue behaved. However, it did not fully capture the unique directional properties of biological tissue. Since the tissue’s mechanical response is directionally dependent, the inclusion of biaxial tensile tests would provide a more comprehensive mechanical characterization.

The model was constructed with a simplified two-layer structure, which does not fully reflect the complexity of the gastric wall. Future models must ideally integrate a more nuanced, multi-layered approach to better represent the contribution of each layer. Another limitation of the current approach is the exclusion of viscoelastic behavior in the model. Biological tissues exhibit time-dependent mechanical responses that were not represented in this study. Incorporating viscoelastic properties into the simulations would better mimic the real-life, dynamic behavior of the stomach tissue under different loading rates.

The current study's stress state mapping did not account for the residual stress that is typically present in tissue. Instead, the simulation highlights the differences from a stress-free condition. The residual stress observed in the samples was limited to the relaxation that happens after the tissue samples are explanted. Additionally, the stress field caused by the needle is expected to be considerably greater than the residual stress field.

The current study employed a manual needle insertion protocol, which introduced potential sources of variability. Manual insertions inherently lack control over the insertion, resulting in potential variability in tissue responses. Misalignment of the needle may alter the force distribution, particularly at the insertion point, potentially skewing mechanical measurements. Standardizing insertions may improve the reproducibility of mechanical measurements near the insertion site. In the current study, the same person did all the insertions and treated all samples similarly. However, in future experiments, automated insertion devices or controlled fixtures will help address these issues better, enabling more consistent and reproducible alignment.

The YAP-1 analysis was limited by the small number of tissue samples obtained from a single pig, with only four samples per test condition. This limited sample size restricted our ability to account for inter-animal and interspecies variability, which would be crucial for robust statistical analysis. Future studies must aim to obtain samples from multiple animals and increase the number of replicates to enhance the results' statistical power and reliability.

## Conclusion

This study provides a validated mechanobiological model linking tissue mechanics and cellular responses to gastric needle insertion. Both experimental and numerical findings highlight a distance-dependent stiffness behavior, with higher stiffness near the needle insertion site, influenced by needle geometry. Cellular-level analysis revealed how mechanical stresses from needle insertion propagate within the tissue, affecting both cytoplasmic and nuclear stress distributions. While YAP-1 and stress expressions show a distance-dependent behavior, the model suggests that sustained or larger-scale mechanical cues are likely needed to trigger mechanotransducive pathways.

These insights can guide studies with more stringent study design and methodology. On a future aspect, these insights may also guide the design of minimally invasive gastric drug delivery devices that aim to minimize tissue disruption while optimizing therapeutic outcomes.

## Supplementary Information

Supplementary file 1

Supplementary file 2

Supplementary file 3

## Data Availability

The data supporting this study's findings are available from the corresponding author upon reasonable request.

## References

[CR1] Abramson A et al (2019a) A luminal unfolding microneedle injector for oral delivery of macromolecules. Nat Med 25:1512–1518. 10.1038/s41591-019-0598-9 31591601 10.1038/s41591-019-0598-9 PMC7218658

[CR2] Abramson A et al (2019b) An ingestible self-orienting system for oral delivery of macromolecules. Science 363:611–615. 10.1126/science.aau2277 30733413 10.1126/science.aau2277 PMC6430586

[CR3] Abramson A et al (2022a) Oral delivery of systemic monoclonal antibodies, peptides and small molecules using gastric auto-injectors. Nat Biotechnol 40:103–109. 10.1038/s41587-021-01024-0 34462588 10.1038/s41587-021-01024-0 PMC8766875

[CR4] Abramson A et al (2022b) Oral mRNA delivery using capsule-mediated gastrointestinal tissue injections. Matter 5:975–987. 10.1016/j.matt.2021.12.022

[CR5] Al-Nuaimi DA et al (2024) Hydrostatic pressure drives sprouting angiogenesis via adherens junction remodelling and YAP signalling. Commun Biol 7:940. 10.1038/s42003-024-06604-9 39097636 10.1038/s42003-024-06604-9 PMC11297954

[CR6] Anselmo AC, Gokarn Y, Mitragotri S (2019) Non-invasive delivery strategies for biologics. Nat Rev Drug Discov 18:19–40. 10.1038/nrd.2018.183 30498202 10.1038/nrd.2018.183

[CR7] Arnold KM, Sicard D, Tschumperlin DJ, Westendorf JJ (2023) Atomic force microscopy micro-indentation methods for determining the elastic modulus of murine articular cartilage. Sensors (Basel) 23:1835. 10.3390/s23041835 36850434 10.3390/s23041835 PMC9967621

[CR8] Bellinger AM et al (2016) Oral, ultra-long-lasting drug delivery: application toward malaria elimination goals. Sci Transl Med 8:365ra157. 10.1126/scitranslmed.aag2374 10.1126/scitranslmed.aag2374 PMC5264553 27856796

[CR20] Brüel A, Christensen EI, Eld M, Prætorius J, Qvortrup K, Tranum-Jensen J, Villadsen R, Geneser F (2020) Genesers histologi. Munksgaard, Danmark

[CR9] Caffarel-Salvador E, Abramson A, Langer R, Traverso G (2017) Oral delivery of biologics using drug–device combinations. Curr Opin Pharmacol 36:8–13. 10.1016/j.coph.2017.07.003 28779684 10.1016/j.coph.2017.07.003 PMC5732838

[CR10] COMSOL (2025) Parameter estimation of hyperelastic materials. Comsol Application Gallary, https://www.comsol.com/model/parameter-estimation-of-hyperelastic-materials-112461

[CR11] Dhalla AK et al (2022) A robotic pill for oral delivery of biotherapeutics: safety, tolerability, and performance in healthy subjects. Drug Deliv Transl Res 12:294–305. 10.1007/s13346-021-00938-1 33604838 10.1007/s13346-021-00938-1 PMC8677648

[CR12] Dou Y, Fan Y, Zhao J, Gregersen H (2006) Longitudinal residual strain and stress-strain relationship in rat small intestine. Biomed Eng Online 5:37. 10.1186/1475-925X-5-37 16759387 10.1186/1475-925X-5-37 PMC1524771

[CR13] Dupont S et al (2011) Role of YAP/TAZ in mechanotransduction. Nature 474:179–183. 10.1038/nature10137 21654799 10.1038/nature10137

[CR14] Elosegui-Artola A et al (2017) Force triggers YAP nuclear entry by regulating transport across nuclear pores. Cell 171:1397-1410.e14. 10.1016/j.cell.2017.10.008 29107331 10.1016/j.cell.2017.10.008

[CR15] Eroumé KS, Cavill R, Staňková K, de Boer J, Carlier A (2021) Exploring the influence of cytosolic and membrane FAK activation on YAP/TAZ nuclear translocation. Biophys J 120:4360–4377. 10.1016/j.bpj.2021.09.009 34509508 10.1016/j.bpj.2021.09.009 PMC8553670

[CR16] Franklin JM, Ghosh RP, Shi Q, Reddick MP, Liphardt JT (2020) Concerted localization-resets precede YAP-dependent transcription. Nat Commun 11:4581. 10.1038/s41467-020-18368-x 32917893 10.1038/s41467-020-18368-x PMC7486942

[CR17] Friis SJ, Hansen TS, Poulsen M, Gregersen H, Nygaard JV (2022) Dynamic viscoelastic properties of porcine gastric tissue: effects of loading frequency, region and direction. J Biomech 143:111302. 10.1016/j.jbiomech.2022.111302 36126503 10.1016/j.jbiomech.2022.111302

[CR18] Friis SJ, Hansen TS, Poulsen M, Gregersen H, Brüel A, Vinge NJ (2023) Biomechanical properties of the stomach: a comprehensive comparative analysis of human and porcine gastric tissue. J Mech Behav Biomed Mater 138:105614. 10.1016/j.jmbbm.2022.105614 36527978 10.1016/j.jmbbm.2022.105614

[CR19] Friis SJ, Hansen TS, Olesen C, Poulsen M, Gregersen H, Vinge NJ (2025) Experimental and numerical study of solid needle insertions into human stomach tissue. J Mech Behav Biomed Mater 162:106832. 10.1016/j.jmbbm.2024.106832 39591721 10.1016/j.jmbbm.2024.106832

[CR21] Ghagre A, Delarue A, Srivastava LK, Koushki N, Ehrlicher A (2024) Nuclear curvature determines Yes-associated protein localization and differentiation of mesenchymal stem cells. Biophys J 123:1222–1239. 10.1016/j.bpj.2024.04.008 38605521 10.1016/j.bpj.2024.04.008 PMC11140468

[CR22] Gregersen H (2000) Residual strain in the gastrointestinal tract: a new concept. Neurogastroenterol Motil 12:411–414. 10.1046/j.1365-2982.2000.00216.x 11012940 10.1046/j.1365-2982.2000.00216.x

[CR23] Guerci B, Chanan N, Kaur S, Jasso-Mosqueda JG, Lew E (2019) Lack of treatment persistence and treatment nonadherence as barriers to glycaemic control in patients with type 2 diabetes. Diabetes Ther 10:437–449. 10.1007/s13300-019-0590-x 30850934 10.1007/s13300-019-0590-x PMC6437240

[CR24] Hashim M, Korupolu R, Syed B, Horlen K, Beraki S, Karamchedu P, Dhalla AK, Ruffy R, Imran M (2019) Jejunal wall delivery of insulin via an ingestible capsule in anesthetized swine—a pharmacokinetic and pharmacodynamic study. Pharmacol Res Perspect 7:e00522. 10.1002/prp2.522 31584244 10.1002/prp2.522 PMC6775958

[CR25] Holzer CS, Pukaluk A, Viertler C, Regitnig P, Caulk AW, Eschbach M, Contini EM, Holzapfel GA (2024) Biomechanical characterization of the passive porcine stomach. Acta Biomater 173:167–183. 10.1016/j.actbio.2023.11.008 37984627 10.1016/j.actbio.2023.11.008

[CR26] Hong W, Guan K-L (2012) The YAP and TAZ transcription coactivators: key downstream effectors of the mammalian Hippo pathway. Semin Cell Dev Biol 23:785–793. 10.1016/j.semcdb.2012.05.004 22659496 10.1016/j.semcdb.2012.05.004 PMC3459069

[CR27] Huang M et al (2023) YAP at the crossroads of biomechanics and drug resistance in human cancer. Int J Mol Sci 24:12491. 10.3390/ijms241512491 37569866 10.3390/ijms241512491 PMC10419175

[CR28] Janmey PA, Wells RG, Assoian RK, McCulloch CA (2013) From tissue mechanics to transcription factors. Differentiation 86:112–120. 10.1016/j.diff.2013.07.004 23969122 10.1016/j.diff.2013.07.004 PMC4545622

[CR29] Janmey PA, Fletcher DA, Reinhart-King CA (2020) Stiffness sensing by cells. Physiol Rev 100:695–724. 10.1152/physrev.00013.2019 31751165 10.1152/physrev.00013.2019 PMC7276923

[CR30] Katzengold R, Shoham N, Benayahu D, Gefen A (2015) Simulating single cell experiments in mechanical testing of adipocytes. Biomech Model Mechanobiol 14:537–547. 10.1007/s10237-014-0620-6 25212098 10.1007/s10237-014-0620-6

[CR31] Kirtane AR et al (2018) Development of an oral once-weekly drug delivery system for HIV antiretroviral therapy. Nat Commun 9:2. 10.1038/s41467-017-02294-6 29317618 10.1038/s41467-017-02294-6 PMC5760734

[CR32] Lin C, Xie J, Li W (2020) Measuring the micromechanical properties of oesophageal mucosa with atomic force microscopy. Biosurf Biotribol 6:97–103. 10.1049/bsbt.2020.0015

[CR33] Liu J et al (2017) Triggerable tough hydrogels for gastric resident dosage forms. Nat Commun 8:124. 10.1038/s41467-017-00144-z 28743858 10.1038/s41467-017-00144-z PMC5527117

[CR34] Liu S, Han Y, Kong L, Wang G, Ye Z (2024) Atomic force microscopy in disease-related studies: exploring tissue and cell mechanics. Microsc Res Tech 87:660–684. 10.1002/jemt.24471 38063315 10.1002/jemt.24471

[CR35] Luo Q, Kuang D, Zhang B, Song G (2016) Cell stiffness determined by atomic force microscopy and its correlation with cell motility. Biochim Biophys Acta 1860:1953–1960. 10.1016/j.bbagen.2016.06.010 27288584 10.1016/j.bbagen.2016.06.010

[CR36] Moroz E, Matoori S, Leroux J-C (2016) Oral delivery of macromolecular drugs: where we are after almost 100years of attempts. Adv Drug Deliv Rev 101:108–121. 10.1016/j.addr.2016.01.010 26826437 10.1016/j.addr.2016.01.010

[CR37] Novev JK, Heltberg ML, Jensen MH, Doostmohammadi A (2021) Spatiotemporal model of cellular mechanotransduction via Rho and YAP. Integr Biol (Camb) 13:197–209. 10.1093/intbio/zyab012 34278428 10.1093/intbio/zyab012

[CR38] Panciera T, Azzolin L, Cordenonsi M, Piccolo S (2017) Mechanobiology of YAP and TAZ in physiology and disease. Nat Rev Mol Cell Biol 18:758–770. 10.1038/nrm.2017.87 28951564 10.1038/nrm.2017.87 PMC6192510

[CR39] Patel B, Gizzi A, Hashemi J, Awakeem Y, Gregersen H, Kassab G (2022) Biomechanical constitutive modeling of the gastrointestinal tissues: a systematic review. Mater des 217:110576. 10.1016/j.matdes.2022.110576 35935127 10.1016/j.matdes.2022.110576 PMC9351365

[CR40] Pocaterra A, Romani P, Dupont S (2020) YAP/TAZ functions and their regulation at a glance. J Cell Sci 133:jcs230425. 10.1242/jcs.230425 31996398 10.1242/jcs.230425

[CR41] Poduval P, Parsekar S, Meena SN (2023) Chapter 10. Small molecules vs biologics. In: Meena SN, Nandre V, Kodam K, Meena RS (eds) New horizons in natural compound research. Academic Press, Houston, pp 179–199. 10.1016/B978-0-443-15232-0.00001-1

[CR42] Poh Y-C, Shevtsov SP, Chowdhury F, Wu DC, Na S, Dundr M, Wang N (2012) Dynamic force-induced direct dissociation of protein complexes in a nuclear body in living cells. Nat Commun 3:866. 10.1038/ncomms1873 22643893 10.1038/ncomms1873 PMC3388544

[CR43] Reddy JN (2013) An introduction to continuum mechanics, 2nd edn. Cambridge University Press, Cambridge. 10.1017/CBO9781139178952

[CR44] Scott KE, Fraley SI, Rangamani P (2021) A spatial model of YAP/TAZ signaling reveals how stiffness, dimensionality, and shape contribute to emergent outcomes. Proc Natl Acad Sci 118:e2021571118. 10.1073/pnas.2021571118 33990464 10.1073/pnas.2021571118 PMC8157940

[CR45] Sferrazza C, Wahlsten A, Trueeb C, D’Andrea R (2019) Ground truth force distribution for learning-based tactile sensing: A finite element approach. IEEE Access 7:173438–173449. 10.1109/ACCESS.2019.2956882

[CR46] Shamir M, Bar-On Y, Phillips R, Milo R (2016) Timescales in cell biology. Cell 164:1302-1302.e1. 10.1016/j.cell.2016.02.058 26967295 10.1016/j.cell.2016.02.058

[CR47] Taheri M, Sousanabadi FA (2024) Experimental extraction of Young’s modulus of gastric tissue with development of spherical, cylindrical, and crowned rollers contact theories. Heliyon 10:e31848. 10.1016/j.heliyon.2024.e31848 38867961 10.1016/j.heliyon.2024.e31848 PMC11167291

[CR48] Tajik A et al (2016) Transcription upregulation via force-induced direct stretching of chromatin. Nat Mater 15:1287–1296. 10.1038/nmat4729 27548707 10.1038/nmat4729 PMC5121013

[CR49] Totaro A, Panciera T, Piccolo S (2018) YAP/TAZ upstream signals and downstream responses. Nat Cell Biol 20:888–899. 10.1038/s41556-018-0142-z 30050119 10.1038/s41556-018-0142-z PMC6186418

[CR50] Traverso G, Schoellhammer CM, Schroeder A, Maa R, Lauwers GY, Polat BE, Anderson DG, Blankschtein D, Langer R (2015) Microneedles for drug delivery via the gastrointestinal tract. J Pharm Sci 104:362–367. 10.1002/jps.24182 25250829 10.1002/jps.24182 PMC4349565

[CR51] Urquhart L (2019) Top drugs and companies by sales in 2018, Nature Reviews Drug Discovery. Gale OneFile: Health and Medicine. link.gale.com/apps/doc/A617024788/HRCA?u=anon~9c706ad&sid=googleScholar&xid=e69a513f. Accessed 6 Aug 2025 10.1038/d41573-019-00049-0 30936513

[CR52] Venturini V et al (2020) The nucleus measures shape changes for cellular proprioception to control dynamic cell behavior. Science 370:eaba2644. 10.1126/science.aba2644 33060331 10.1126/science.aba2644

[CR53] Verma M et al (2019) A gastric resident drug delivery system for prolonged gram-level dosing of tuberculosis treatment. Sci Transl Med 11:eaau6267. 10.1126/scitranslmed.aau6267 30867322 10.1126/scitranslmed.aau6267 PMC7797620

[CR54] Zheng Y et al (2013) Shear wave propagation in soft tissue and ultrasound vibrometry. In: Wave propagation theories and applications, IntechOpen.10.5772/48629

[CR55] Zimmerli CE et al (2021) Nuclear pores dilate and constrict in cellulo. Science 374:eabd9776. 10.1126/science.abd9776 34762489 10.1126/science.abd9776

