# Supplementary material for: Mechanobiology of gastric needle insertions: a combined experimental and numerical study
Source: Biomech Model Mechanobiol. 2025 Aug 13;24(5):1633–51. doi: 10.1007/s10237-025-01986-z (PMC12454536; doi:10.1007/s10237-025-01986-z)
Supplement: Supplementary file 3 [file 10237_2025_1986_MOESM3_ESM.docx]

# Supplementary Materials

## Constitutive parameters from fitting procedure to experimental tension and radial compression tests

|  | **Longitudinal**  **tension** | | **Circumferential**  **tension** | | **Radial**  **compression** | | |
| --- | --- | --- | --- | --- | --- | --- | --- |
|  | **σ_T_(λ)=b∙(exp(a∙ (λ-1))-1)** | | | | **σ_C_(λ)=2∙ (λ - λ ^-2^) ∙**  **(C_10_+C_01_∙x^-1^+2∙C_20_∙ ( λ ^2^+2∙x^-1^-3))** | | |
|  | a | b | a | b | C_10_ | C_01_ | C_20_ |
| **Mean** | 20.6 | 0.3 | 14.8 | 0.6 | -15.5 | 17.9 | 13.1 |
| **STD** | 2.4 | 0.2 | 1.7 | 0.3 | 3.6 | 4.0 | 3.0 |
| **Min.** | 17.3 | 0 | 12.1 | 0.2 | -20.0 | 12.2 | 9.1 |
| **Q1** | 19.2 | 0.2 | 13.6 | 0.4 | -17.7 | 16.8 | 12.1 |
| **Median** | 21.4 | 0.3 | 15.0 | 0.4 | -15.2 | 17.4 | 12.2 |
| **Q3** | 21.6 | 0.3 | 15.8 | 0.9 | -14.5 | 20.3 | 15.2 |
| **Max.** | 24.1 | 0.6 | 17.3 | 1.1 | -10.4 | 22.8 | 16.7 |

Table S- 1. Replication of experimental results from Friis et al. [34] used in equation 7.

## Anderson-Darling normality test of AFM indentation data

The data were considered normally distributed for p>0.05.

|  | **Blunt** | **Sharp** |
| --- | --- | --- |
| **Point** | p-value | p-value |
| **0** | 0.2187 | 0.0204 |
| **1** | 0.6401 | 0.00 |
| **2** | 0.3786 | 0.00 |
| **3** | 0.0031 | 0.00 |
| **4** | 0.00 | 0.05 |
| **5** | 0.00 | 0.06 |
| **6** | 0.00 | 0.53 |
| **7** | 0.00 | 0.00 |
| **8** | 0.33 | 0.00 |
| **9** | 0.12 | 0.01 |
| **11** | 0.00 | 0.07 |
| **14** | 0.00 | 0.00 |
| **18** | 0.05 | 0.00 |

Table S- 2

## Table S-1 - Blunt AFM measures

| **Point** | **0** | **1** | **2** | **3** | **4** | **5** | **6** | **7** | **8** | **9** | **11** | **14** | **18** |
| --- | --- | --- | --- | --- | --- | --- | --- | --- | --- | --- | --- | --- | --- |
| **Mean** | 57.93 | 47.52 | 65.06 | 41.55 | 56.66 | 29.80 | 34.17 | 50.52 | 31.11 | 25.15 | 19.64 | 50.86 | 37.26 |
| **SD** | 28.92 | 9.67 | 21.23 | 20.45 | 24.24 | 14.40 | 18.62 | 20.38 | 6.00 | 8.49 | 16.42 | 43.65 | 6.21 |
| **Min.** | 17.09 | 33.70 | 18.31 | 11.75 | 18.71 | 18.12 | 15.72 | 25.49 | 17.93 | 8.37 | 6.43 | 4.11 | 24.42 |
| **Q1** | 37.90 | 40.46 | 52.75 | 20.04 | 24.39 | 20.86 | 22.07 | 34.19 | 28.43 | 21.42 | 12.69 | 16.44 | 32.96 |
| **Q2** | 51.61 | 48.23 | 62.55 | 53.41 | 72.61 | 24.20 | 31.89 | 44.41 | 31.94 | 25.77 | 13.94 | 39.13 | 39.41 |
| **Q3** | 35.99 | 54.87 | 77.85 | 57.89 | 75.29 | 33.81 | 38.09 | 64.60 | 34.97 | 29.95 | 16.91 | 93.74 | 41.54 |
| **Max.** | 30.73 | 62.46 | 113.76 | 68.68 | 81.71 | 79.55 | 115.99 | 94.53 | 41.41 | 46.37 | 82.84 | 131.19 | 44.08 |

Table S- 3. Statistical measures from AFM indentations for samples with a blunt needle tip geometry inserted. n=10 samples were tested, and in total N=10 indentations were done in each point.

## Table S-2 - Sharp AFM measures

| **Point** | **0** | **1** | **2** | **3** | **4** | **5** | **6** | **7** | **8** | **9** | **11** | **14** | **18** |
| --- | --- | --- | --- | --- | --- | --- | --- | --- | --- | --- | --- | --- | --- |
| **Mean** | 41.49 | 41.33 | 28.78 | 35.27 | 32.20 | 40.88 | 31.65 | 56.24 | 32.07 | 44.87 | 25.23 | 16.25 | 30.44 |
| **SD** | 20.72 | 40.93 | 13.39 | 26.26 | 9.76 | 11.20 | 9.03 | 15.67 | 11.01 | 19.18 | 10.78 | 13.19 | 21.92 |
| **Min.** | 18.08 | 10.13 | 13.41 | 13.52 | 15.51 | 14.52 | 11.36 | 14.67 | 7.32 | 11.94 | 6.10 | 2.09 | 4.80 |
| **Q1** | 28.61 | 17.00 | 17.80 | 17.04 | 25.27 | 33.19 | 26.16 | 49.28 | 29.72 | 31.21 | 17.02 | 7.79 | 15.54 |
| **Q2** | 33.29 | 27.67 | 22.97 | 25.30 | 35.08 | 42.63 | 29.46 | 52.05 | 34.00 | 52.90 | 23.38 | 11.70 | 21.54 |
| **Q3** | 50.31 | 47.81 | 36.93 | 37.56 | 39.75 | 45.50 | 37.98 | 61.27 | 40.74 | 60.18 | 35.79 | 18.01 | 50.73 |
| **Max.** | 91.62 | 200.25 | 56.31 | 94.89 | 43.62 | 68.28 | 52.73 | 97.77 | 42.13 | 65.56 | 50.73 | 51.38 | 71.89 |

Table S- 4. Statistical measures from AFM indentations for samples with a sharp needle tip geometry inserted. n=10 samples were tested,.and in total N=10 indentations were done in each point.

## R^2^ values and P-values from linear regression. F-test is testing if stiffness measured in AFM indentation is distance-dependent

p>0.05 indicates that the distance-dependent stiffness behavior is following a linear line.

|  | **Blunt** | | **Sharp** | |
| --- | --- | --- | --- | --- |
|  | Mean | Median | Mean | Median |
| **F-test (p-value)** | 0.1228 | 0.0895 | 0.1666 | 0.06237 |
| **R^2^** | 0.2025 | 0.2397 | 0.1663 | 0.4106 |

Table S- 5

## ANCOVA p-values from linear regression testing if slope and intercepts of blunt and sharp needles are different from each other (AFM indentation)

P<0.05 indicates that slope and intercepts are different from each other for blunt and sharp needle

|  | **Slope** | | **Intercept** | |
| --- | --- | --- | --- | --- |
|  | Mean | Median | Mean | Median |
| **ANCOVA**  **(p-value)** | 0.6440 | 0.3741 | 0.1261 | 0.0779 |

Table S- 6

## P-values from t-test / Wilcoxon rank sum test testing is stiffness measured in AFM indentation point 0 and 18 significantly differ

p<0.05 indicates a difference between the stiffness measured in the two indentation points.

|  | **Blunt** | **Sharp** |
| --- | --- | --- |
| **T-test**  **(p-value)** | 0.0003 | 0.0655 |
| **Wilcoxon rank sum test**  **(p-value)** | 0.0213 | 0.0110 |

Table S- 7

## R^2^ values and P-values from linear regression. F-test is testing if YAP-1 fractions measured is distance-dependent

p>0.05 indicates that the YAP-1 fraction distance-dependent behavior is following a linear line.

|  | **Blunt** | **Sharp** |
| --- | --- | --- |
| **F-test (p-value)** | 0.0049 | 0.2605 |
| **R^2^** | 0.3073 | 0.07381 |

Table S- 8

## ANCOVA p-values from linear regression testing if slope and intercepts of blunt and sharp needles are different from each other (YAP-1 fraction)

P<0.05 indicates that slope and intercepts are different from each other for blunt and sharp needle

|  | **Slope** | **Intercept** |
| --- | --- | --- |
| **ANCOVA**  **(p-value)** | 0.2098 | 0.6722 |

Table S- 9
